# Pharmacokinetics and safety of cyclophosphamide and docetaxel in a hemodialysis patient with early stage breast cancer: a case report

**DOI:** 10.1186/s12885-015-1932-3

**Published:** 2015-11-18

**Authors:** Liu Yang, Xiao-chen Zhang, Su-feng Yu, Hua-Qing Zhu, Ai-ping Hu, Jian Chen, Peng Shen

**Affiliations:** 1 Department of Medical oncology, the First Affiliated Hospital, College of Medicine, Zhejiang University, Qingchun Road 79#, Hangzhou, Zhejiang 310003 People’s Republic of China; 2 Department of Pharmacy, the First Affiliated Hospital, College of Medicine, Zhejiang University, Qingchun Road 79#, Hangzhou, Zhejiang 310003 People’s Republic of China; 3 Department of Medical Oncology, Zhejiang Provincial People’s Hospital, Hangzhou, 310014 People’s Republic of China

**Keywords:** Cyclophosphamide, Docetaxel, Pharmacokinetics, Hemodialysis, Breast cancer

## Abstract

**Background:**

Standardized chemotherapy used in cancer patients with severe kidney insufficiency is ineffective. Although there are some pharmacokinetic studies on cyclophosphamide in kidney insufficiency patients, to the best of our knowledge, the pharmacokinetics and safety of combination of cyclophosphamide and docetaxel as postoperative chemotherapy in a patient with early stage breast cancer undergoing hemodialysis is unclear thus far.

**Case Presentation:**

The patient received regular TC regimen (cyclophosphamide 600 mg/m^2^, docetaxel 75 mg/m^2^). She underwent hemodialysis 48 h after chemotherapy. Blood samples at multiple time-points were collected for determination of plasma levels of cyclophosphamide and docetaxel. Pharmacokinetic analyses indicated that compared with the reference data, the in vivo half-life (66.96 h) and drug exposure (150 %) of cyclophosphamide significantly increased; however, pharmacokinetic parameters of docetaxel was unaffected. Patient developed grade I thrombocytopenia and grade III leukopenia without any other severe adverse reactions. In total, four cycles of treatment were completed. After the chemotherapy, the patient received tamoxifen as endocrine therapy for one and a half years. No recurrence was reported.

**Conclusion:**

These results suggest that the standard TC regimen is mostly safe and could be used as postoperative adjuvant chemotherapy for hemodialysis patients with early stage breast cancer.

## Background

Cancer patients with chronic kidney disease are not uncommon. These patients generally have a high mortality. The mortality rate of a cancer patient increases by 22 % when the estimated glomerular filtration rate (GFR) decreases to 10 mL/min/1.73 m^2^ [1, 2]. This finding can be attributed to the fact that GFR reduction not only induces severe kidney complications but also restricts tumor treatment, thereby promoting tumor progression.

GFR reduction reduces drug excretion and thus enhances drug exposure. This phenomenon leads to increased toxicity, particularly in patients with severe kidney insufficiency (phase III and above). Thus, those patients with chemotherapy indications would have few treatment options. Therefore, cancer patients with kidney insufficiency are subject to high risks of tumor recurrence and metastasis. However, whether or not such patients can receive standardized chemotherapy or whether dose adjustments are required for these patients, particularly those with end-stage renal disease (ESRD), remains unknown thus far.

In this study, a patient with mastocarcinoma accompanied by ESRD received postoperative cyclophosphamide/docetaxel (TC) regimen. The peripheral blood levels of these two drugs were measured to evaluate their pharmacokinetics, efficacy, and safety in the patient. TC (600 mg/m^2^ cyclophosphamide + 75 mg/m^2^ docetaxel) is a treatment regimen recommended by the National Comprehensive Cancer Network Guide to Practice of Breast Cancer 2014 ver 1 [3]. Up to 50 % to 70 % of cyclophosphamide is eliminated through the kidney within 48 h with 32 % eliminated unchanged and 68 % eliminated as metabolites. Docetaxel and its metabolites are eliminated in feces (75 %) and urine (6 %) within 48 h after administration [4, 5]. It is obvious that both cyclophosphamide and docetaxel are influenced by kidney excretory function, particularly cyclophosphamide. Elimination distribution ratio is altered (e.g., improved stool excretion) when chemotherapy drugs get blocked in the main drainage channel. In addition, the plasma concentration of chemotherapy drugs is possibly inconsistent with the side effect of treatment. Thus, the feasibility of using the TC regimen as adjuvant chemotherapy in a hemodialysis patient with early stage breast cancer warrants further investigation.

## Case Presentation

### Case description

A 48-year-old female patient underwent modified left radical mastectomy, pT_2_N_0_M_0_, IIa stage. Pathological examination revealed infiltrative duct carcinoma (Level II according to WHO classification) that was estrogen receptor-positive (ER+, 95 %), progesterone receptor-positive (PR+, 85–90 %), C-erB_2_ (1+), and Ki-67 (+, 35 %).

The patient had uremia eight years ago. She received conventional hemodialysis treatment for eight years (hemodialysis every 48 h), and has produced no urine. The patient was administered 3000 units of erythropoietin (biw) for chronic kidney disease accompanied by mild to moderate anemia. The patient was diagnosed with hypertension eight years ago. She was taking one pill of nifedipine (bid) and 12.5 mg of metoprolol (bid) to manage her blood pressure.

The treatment protocol for the patient was approved by the Ethics Committee of the First Affiliated Hospital, School of Medicine, Zhejiang University, and written informed consent was obtained from her.

### Treatment and Hemodialysis

After the operation, the patient (body surface area, 1.50 m^2^) received 113 mg of 75 mg/m^2^ TC docetaxel (ivgtt, day 1) and 900 mg of 600 mg/m^2^ cyclophosphamide (ivgtt, day 1). The drugs were administered 2 h after hemodialysis. Cyclophosphamide was administered by intravenous drip infusion over 60 min. Then, normal saline was administered for 10 min for catheter rinsing. Docetaxel was then administered by intravenous drip infusion over 50 min. After 48 h, the next hemodialysis was performed with Polyflux 14 L (Corp Gambro, Germany). The UF coefficient in vitro and membrane area were 10.0 ml/(h · mmHg) and 1.4 m^2^, respectively. The duration of hemodialysis was 4 h. This program was repeated every three weeks, for a total of four cycles. White blood cell count, blood platelet count, hemoglobin count, liver and kidney functions, and other indicators were regularly monitored during the treatment process. When grade III neutropenia occurred, short-term granulocyte colony stimulating factor (GCSF) was administered to support the treatment.

### Pharmacokinetic analysis

We investigated the pharmacokinetics of docetaxel and cyclophosphamide. A 5-mL aliquot of peripheral blood was collected before administration of the regimen, 0.5, 1, 1.5, 2, 4, 6, 8, 12, 24, 36, and 48 h after cyclophosphamide treatment, and 2 h after hemodialysis. The blood was stored at 4 °C by using EDTA as an anticoagulant. Then, the blood was centrifuged at 8000 rpm for 10 min. Plasma was stored at −80 C for later analyses. Agilent 6460 Triple quadrupole liquid chromatography-mass spectrometer (LC-MS/MS) (Agilent, Palo Alto, CA) was used as previously described [6, 7] to measure the plasma concentrations of cyclophosphamide and docetaxel. The linear correlation coefficient (r^2^), limit of detection, and limit of quantitation for cyclophosphamide were 0.994, 0.5 μg/mL, and 1.5 μg/mL, respectively, whereas those for doectaxel were 0.994, 0.1 ng/mL, and 0.5 ng/mL, respectively.

The data were analyzed using descriptive PK methods by employing Kinetica version 5.0 (ThermoFisher Scientific, CO, USA). The PK parameters of cyclophosphamide and docetaxel were estimated using non-compartmental method and standard two-compartmental method based on the plasma concentrations excluding the post-dialysis sample, respectively, and compared with the data reported in the literature from patients with normal kidney function (for cyclophosphamide, compared with literature [8, 9], for docetaxel, compared with literature [10], [11], and [12]).

## Results

**Fig. 1:**
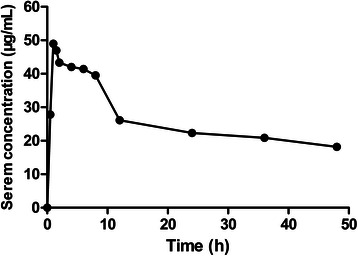
The serum concentration-time curves of cyclophosphamide

**Fig. 2:**
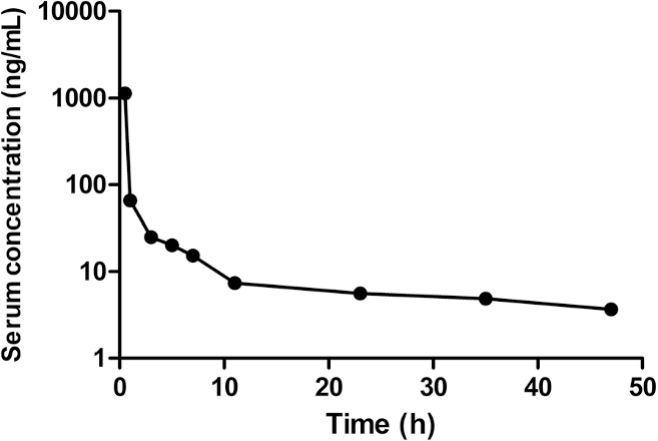
The serum concentration-time curves of docetaxel

The pharmacological characteristics of the patient administered cyclophosphamide and docetaxel are shown in Fig. 1, Fig. 2, and Table 1. The maximum plasma concentration of cyclophosphamide was 48.97 μg/mL. After non-compartmental model fitting, the area under the curve (AUC_0~∞_) was 3128 μg · h/mL and the in vivo half-life was 66.96 h (Fig. 1 and Table 1). These values were 1.5- and 11-fold higher than the typical values of the reference group, respectively. The two-compartment model was the best-fit model of docetaxel pharmacokinetics. The highest plasma concentration, AUC_0~∞,_ and in vivo half-life of decetaxel were 65.85 ng/mL, 4430 ng · h/mL, and 21.53 h, respectively; these values were similar to the typical values of the reference group (Fig. 2 and Table 1). After hemodialysis, the plasma concentrations of cyclophosphamide and docetaxel decreased by 1.25 μg/mL (6.9 %) and 0.11 ng/mL (2.9 %), respectively.

**Table 1:** Pharmacokinetic parameters of the analyzed patient and reported data of patients with normal kidney function

Compound	Unit	Patient	Reference	Deviation (%)
Cyclophosphamide				
C_max_	μg/mL	48.97	61.32	21.2 %
t_1/2_	hour	66.96	4 ~ 6	1016 %
AUC_0~∞_	μg · h/mL	3128	1917	56 %
Docetaxel				
C_max_	ng/mL	1132.6	1309 ~ 2930	13.6 ~ 61.4 %
t_1/2_	hour	21.53	7.8 ~ 21.0	2.5 ~ 176 %
AUC_0~∞_	ng · h/mL	4430	1990 ~ 3770	17 ~ 122 %

Four cycles of treatment were smoothly completed. The patient developed grade I thrombocytopenia and grade III leucopenia (classified according to NCI Common Terminology Criteria for Adverse Events Version 4) after chemotherapy. These conditions improved after administering suitable treatments. A previous study reported that the incidence rates of grade III leukopenia and grade I thrombocytopenia are approximately 10 % and 1 %, respectively, when the TC regimen is used for breast cancer [13]. After chemotherapy, the patient received tamoxifen as endocrine therapy for one and a half years. No recurrence was reported.

## Conclusions

Kidney insufficiency affects the accumulation of drugs in the body, increases drug exposure, and causes treatment safety issues. Therefore, clinicians take extra cautions when treating cancer patients with moderate to severe kidney insufficiency. Discontinuation of chemotherapy owing to increased side effects decreases the therapeutic effect of a regimen.

This study attempted to use conventional TC regimen as adjuvant chemotherapy to treat a hemodialysis patient with early stage breast cancer. Exposure to cyclophosphamide significantly increased whereas the pharmacokinetics of docetaxel remained unchanged in this patient. Mild hematologic toxicity was observed in the patient without severe non-hematologic toxicity. The patient received four cycles of treatments. No tumor recurrence and metastasis have been reported yet (i.e., after a two-year follow-up period). These data suggest that adjuvant chemotherapy with the standardized dosages of cyclophosphamide and docetaxel is safe and effective when administered postoperatively to breast cancer patients with severe kidney insufficiency.

Several studies of the pharmacokinetics of cyclophosphamide in kidney insufficiency patients have already been reported. Haubitz et al. [14] found that cyclophosphamide exposure increased as the severity of kidney damage increased; for example, the cyclophosphamide exposure increased by 1.2-fold in patients with phase V kidney damage. Ekhart et al. [15] investigated the pharmacokinetic changes of cyclophosphamide in patients with moderate kidney insufficiency. They found that the 67 % increase in cyclophosphamide exposure might be not sufficient to adjust the dosage. However, the changes in the pharmacokinetics of cyclophosphamide in hemodialysis patients remain unclear thus far. In the present study, although drug exposure significantly increased (150 %), the standard dosage of cyclophosphamide was still found to be relatively safe for the mastectomy patient with ESRD. In fact, 1000 mg of cyclophosphamide as an imperative treatment can still be highly tolerated by patients with immune diseases, including lupus nephritis and chronic kidney disease [16]. This finding may partially explain the tolerance to high cyclophosphamide concentrations observed in this study.

Docetaxel is not usually administered to patients with renal insufficiency because of its low excretion rate via the kidneys. Dimopoulos et al. [17] investigated the effects of docetaxel in patients with urinary tract tumors and late-stage kidney damage. They found that the toxic effects of docetaxel slightly increased in patients with kidney damage. However, the study did not investigate the pharmacokinetic characteristics of docetaxel. The present study showed that the pharmacokinetic parameters of docetaxel remained unaffected and were comparable to those in the reference group, thereby meeting the two-compartment model. Doxetaxel exposure slightly increased, but no relative toxic effects on the patient were observed. Similarly, postoperative breast cancer patients with ESRD can adapt to the normal dosage of docetaxel.

Another interesting finding in our study is that hemodialysis did not significantly influence the clearance of cyclophosphamide and docetaxel. There are a few case reports on pharmacokinetics of cyclophosphamide and docetaxel in patients undergoing hemodialysis. Haubitz M et al. [14] studied the effects of hemodialysis on the pharmacokinetics of cyclophosphamide, and proved that the plasma concentration of cyclophosphamide decreased by 10 % after hemodialysis, while Hochegger K et al. [18] revealed that following hemodialysis, the concentration of docetaxel decreased by nearly 92 %. However, in this study, the plasma concentrations of cyclophosphamide and docetaxel decreased by only 6.9 % and 2.9 % after hemodialysis, respectively, probably owing to individual variability of cyclophosphamide clearance and low concentration of docetaxel in plasma at 48 h that led to limited elimination by hemodialysis. Therefore, we speculate that the decreased drug toxicity was not attributed to hemodialysis. However, the white blood cell count of the patient increased to 40.5 × 10^9^/L during the treatment process. This result is attributable to the application of GCSF. As a macromolecular protein with a molecular weight of approximately 20000, GCSF will accumulate in the body because it cannot be completely eliminated by hemodialysis in the present study. The conventional dosage of short-term GCSF might markedly increase white blood cell and neutrophilic granulocyte count. The white cell count of the patient gradually normalized upon discontinuation of GCSF for 1 week. Therefore, the dosage and frequency of GCSF should be adjusted accordingly in hemodialysis patients.

This study has two limitations. First, we did not measure the metabolites of cyclophosphamide and docetaxel owing to unavailability of the standard metabolites required for such analysis, which might explain the lack of published data on such metabolites. Ekhart C et al. demonstrated that the active metabolites of cyclophosphamide were not significantly affected in conditions of renal impairment, similar to the condition described in the present study [6]. Second, the generalizability of the case still needs to be verified. Despite these limitations, this study is the first to present the pharmacokinetic data of cyclophosphamide and docetaxel in a breast cancer patient with ESRD. Overall, the TC regimen is probably safe and could be used for similar cases.

## Consent

Written informed consent was obtained from the patient for publication of this case report. A copy of the written consent is available for review by the editor of this journal.

## Abbreviations

GFR: Glomerular filtration rate
ESRD: End-Stage Renal Disease
TC: Cyclophosphamide/docetaxel
GCSF: Granulocyte colony stimulating factor
LC-MS/MS: Triple quadrupole liquid chromatography-mass spectrometer
